# Effect of pathological heterogeneity on shear wave elasticity imaging in the staging of deep venous thrombosis

**DOI:** 10.1371/journal.pone.0179103

**Published:** 2017-06-14

**Authors:** Xiaona Liu, Na Li, Chaoyang Wen

**Affiliations:** 1 Chinese PLA (People's Liberation Army) Medical School, Beijing, P.R. China; 2 Department of Ultrasound, Binzhou Medical University Hospital, Binzhou, Shandong, P.R. China; 3 Department of Auxiliary Diagnosis, The 463rd Hospital of Shenyang Military Region, Shenyang, Liaoning, P.R. China; 4 Department of Ultrasound, The First Affiliated Hospital of Chinese PLA General Hospital, Beijing, P.R. China; University of Montreal Hospital Research Center, CANADA

## Abstract

**Background:**

We aimed to observe the relationship between the pathological components of a deep venous thrombus (DVT), which was divided into three parts, and the findings on quantitative ultrasonic shear wave elastography (SWE) to increase the accuracy of thrombus staging in a rabbit model.

**Methods:**

A flow stenosis-induced vein thrombosis model was used, and the thrombus was divided into three parts (head, body and tail), which were associated with corresponding observation points. Elasticity was quantified in vivo using SWE over a 2-week period. A quantitative pathologic image analysis (QPIA) was performed to obtain the relative percentages of the components of the main clots.

**Results:**

DVT maturity occurred at 2 weeks, and the elasticity of the whole thrombus and the three parts (head, body and tail) showed an increasing trend, with the Young's modulus values varying from 2.36 ± 0.41 kPa to 13.24 ± 1.71 kPa; 2.01 ± 0.28 kPa to 13.29 ± 1.48 kPa; 3.27 ± 0.57 kPa to 15.91 ± 2.05 kPa; and 1.79 ± 0.36 kPa to 10.51 ± 1.61 kPa, respectively. Significant increases occurred on different days for the different parts: the head showed significant increases on days 4 and 6; the body showed significant increases on days 4 and 7; and the tail showed significant increases on days 3 and 6. The QPIA showed that the thrombus composition changed dynamically as the thrombus matured, with the fibrin and calcium salt deposition gradually increasing and the red blood cells (RBCs) and platelet trabecula gradually decreasing. Significant changes were observed on days 4 and 7, which may represent the transition points for acute, sub-acute and chronic thrombi. Significant heterogeneity was observed between and within the thrombi.

**Conclusions:**

Variations in the thrombus components were generally consistent between the SWE and QPIA. Days 4 and 7 after thrombus induction may represent the transition points for acute, sub-acute and chronic thrombi in rabbit models. A dynamic examination of the same part of the thrombus may be helpful for improving the sensitivity and reproducibility of SWE for DVT diagnosis and staging.

## Introduction

Deep venous thrombosis (DVT) is the third most common vascular disease, and it is primarily caused by the abnormal coagulation of venous reflux. More than 2 million people in the United States are affected each year [1, 2], and an estimated 600,000 patients develop pulmonary embolism (PE) and 60,000 die of related complications each year [1, 3]. Studies have shown that DVT-associated morbidity has been gradually increasing in recent years [4]; thus, the efficient prevention and control of DVT and its fatal complication PE is important.

DVT can be roughly divided into the following three clinical periods: acute, sub-acute and chronic. During these periods, acute thrombi are considered to be prone to detaching from the vein, thus presenting a higher risk for PE. Different treatment strategies are applied at different stages, and patients with acute DVT are treated with heparin followed by oral anticoagulants while patients with chronic thrombi are treated with oral anticoagulants alone[5]. Therefore, accurately estimating the age and maturity of DVT is of great importance for determining therapeutic management.

Venous duplex ultrasonography combines color flow Doppler imaging with compression ultrasonography and is considered the gold standard first-line investigation for imaging DVT [6]. However, this method cannot sufficiently determine the age of DVT [7]. The pathological composition of thrombi changes as the clots mature, and the composition of acute clots, including platelets, fibrin, and neutrophils, is replaced by collagen and mononuclear cells over time [8], which alters the mechanical properties of the blood clot [9]. This transition progressively hardens clots. Ultrasonic elastography is a technique that uses tissue deformation to assess the local hardness of the tissues undergoing deformation. Emelianov et al. [6] applied quasi static elastography to assess the mechanical properties of clots, and found that the age or maturity of thrombi was related with their elasticity. Several studies have demonstrated the value of elastography in staging thrombi as an adjunct to conventional ultrasound (US)[2, 10]. Shear wave elastography (SWE) is a new technique that can assess tissue elasticity based on a linear isotropic mechanical model by measuring the transmission speed of a shear wave. Some researchers have applied SWE to investigate the viscoelasticity features of thrombus[11]. Genisson JL et al.[12] indicated that the mechanical properties of blood clot could be assessed by the transient elastography technique. Bernal M et al.[13] validates the use of supersonic shear wave imaging (SSI) to study the rheologic properties of soft solids as well as materials with changing mechanical properties. SWE can also provide consistent quantitative measurements for diagnosing and following venous thrombosis [14].

Although previous results have demonstrated the value of SWE in staging DVT[14, 15] and found that the foundation for staging is mainly based on changes in rigidity caused by variations in the pathological structure, systematic studies have not been performed to evaluate the relationship between the pathological contents of DVT and the findings on SWE. The mechanisms underlying the formation and development of DVT differ in different parts of the thrombus, and whether these different parts affect the staging process remains unknown.

The purpose of the present study was to observe the relationship among the pathological components of different parts of DVT and the findings on SWE during thrombus maturity to obtain a more accurate picture of the thrombus stages. A quantitative pathologic image analysis (QPIA)[16] was performed to observe the dynamic changes of DVT structures in different areas with thrombus maturity. We further explored the effects of different clot parts for staging thrombus using SWE. Our intention was to provide insights to help guide SWE applications for staging DVT.

## Materials and methods

### Animal preparation

Animal studies were performed using a rabbit model of stenosis-induced venous thrombosis. Fifty-six healthy female Japanese white rabbits weighing 3.1 ± 0.3 kg were used following approval by the Institutional Care & Use Committee (IACUC) of The First Affiliated Hospital of Chinese PLA General Hospital [SYXK(JUN)2012-0014]. All animals were purchased from the laboratory animal department of The First Affiliated Hospital of Chinese PLA General Hospital (Beijing, China). The vendor provided health reports to certify that all rabbits were free of pathogens. All rabbits were individually housed in clear iron cages on a suspended floor under controlled conditions (temperature, 21 ± 2°C; relative humidity, 50 ± 20%; air ventilation, 10 to 15 times per hour; artificial lighting, 12 hours per day). All rabbits received food and water ad libitum and were checked daily to observe their general condition during the experimental period.

### Animal models

All animals were anesthetized via the ear vein with 1 ml/kg 3% pentobarbital sodium (CAS:57-33-0, Merck, Germany), and an additional dose at 50% of the first dose could be administered during the experimental process if necessary. Rabbits were then placed in a supine position on an operating table, and the rectal body temperature was monitored and maintained approximately 38°C.

**Fig 1:**
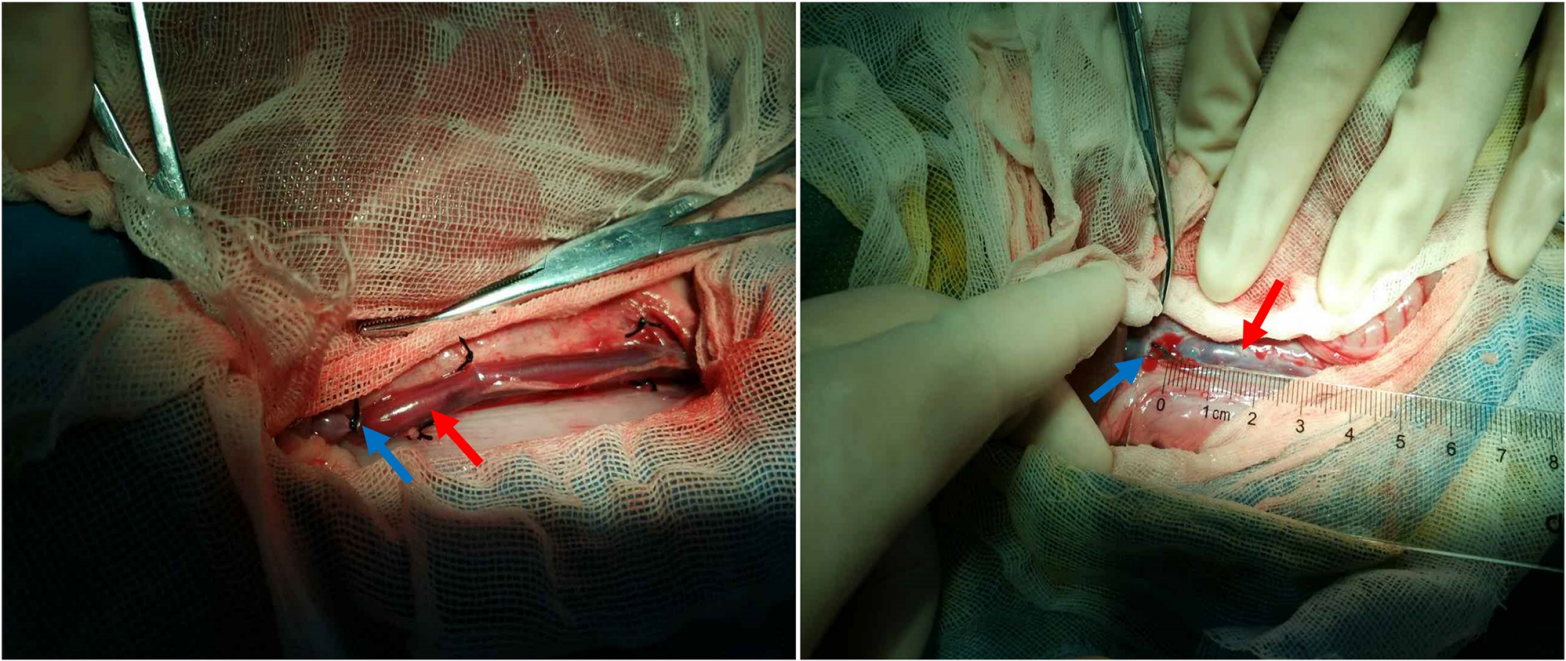
Venous thrombus induced by stenosis models in the rabbit inferior vena cava (IVC). Left: fresh blood clots in the IVC immediately after thrombosis induction. Right: thrombus on day 7 before exteriorizing presenting a dark red color with small patches of calcification. Blue and red arrows indicate the position of the ligation and blood clots, respectively.

A midline laparotomy was performed, and the inferior vena cava (IVC) under the renal veins to the iliac vein bifurcation was chosen as the location of induced thrombus. The interval IVC was exposed and isolated using blunt dissection with all branches ligated. Noninvasive vascular clamps were used to create stasis at both ends for 15 minutes, and the nipped IVC was tonged gently by hemostatic forceps. Then, the proximal location was ligated incompletely with a channel diameter close to that of a #5 syringe needle. Subsequently, the two vascular clamps were removed in sequence. As a direct result, thrombosis was initiated from the location of stenosis and stretched a blood coagulation (Fig 1). US examination was used to evaluate whether clots occurred.

More than ten rabbits were selected each day for elasticity measurements using SWE, and three cases were randomly selected for pathological examination.

### Shear wave elasticity imaging

In the present study, US examinations were performed with the Aixplorer ultrasound system (Supersonic Imagine, Aixen-Provence, France) equipped with an SL 2–10 MHz transducer. During the course of the examination, the probe was used as lightly as possible to avoid applying pressure on the thrombus, and it was remained in a stable position for at least 10 seconds during image acquisition. The vein under analysis and the adjacent tissue were documented, and the region of interest (ROI) was selected for subsequent elasticity data collection. When a stable color-coded elasticity map was obtained, gray-scale and elasticity images were captured according to a standard US appointment.

The ROIs were manually drawn over the selected portion of the thrombus. The mean value of Young's modulus (YM) within the ROIs was recorded by processing the image with the system’s built-in tool for quantifying ROI, which is termed the “Q-Box” (SuperSonic Imagine). The system calculated the Emean, Emin, Emax and standard deviation (SD). In the present study, the Emean and SD were recorded. And the measurement was repeated three times and the mean value was taken as the finally determined YM value. In addition, a color-coded map was obtained with a default color scale ranging from dark blue to deep red representing the lowest to highest stiffness (0–80 kPa). Different stiffness levels in the ROIs were represented with different colors. Measurements were performed in triplicate to ensure the consistency of results.

**Fig 2:**
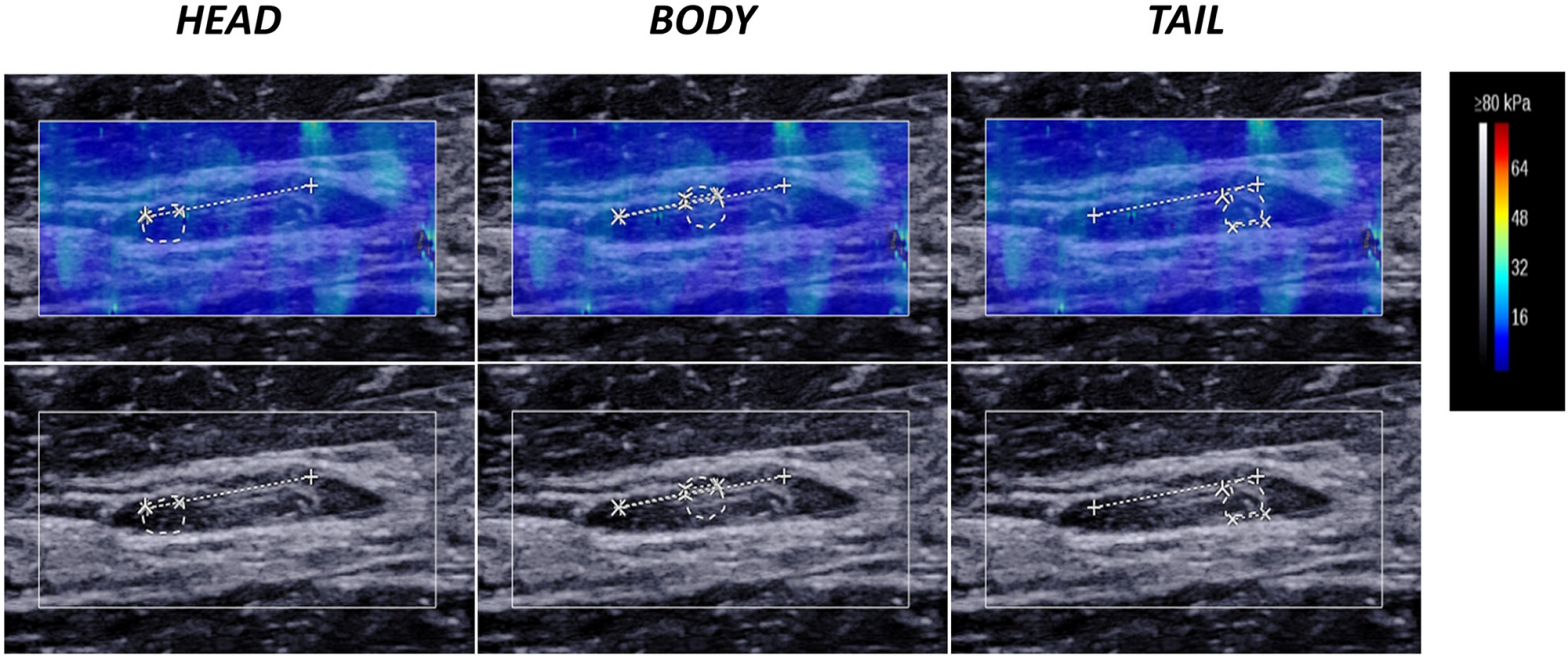
Ultrasound measurement of the thrombus on day 14 in the head, body and tail. The upper and lower row show the shear wave elastography and the gray-scale ultrasound findings of the three parts of the thrombus. The region of interest (ROI) boundary is indicated by a dashed-line circle. As shown in the colour bar, clot hardening correlates with a gradual color change within the clot from dark blue to deep red.

In the present study, we trisected the thrombus as much as possible into the following three parts: head, body and tail. The thrombus-containing IVC was measured in the longitudinal section plane (i.e., parallel to the long axis of the thrombus), and the three parts were measured independently (Fig 2).

### Ex vivo tissue samples and quantitative pathologic image analysis (QPIA)

**Fig 3:**
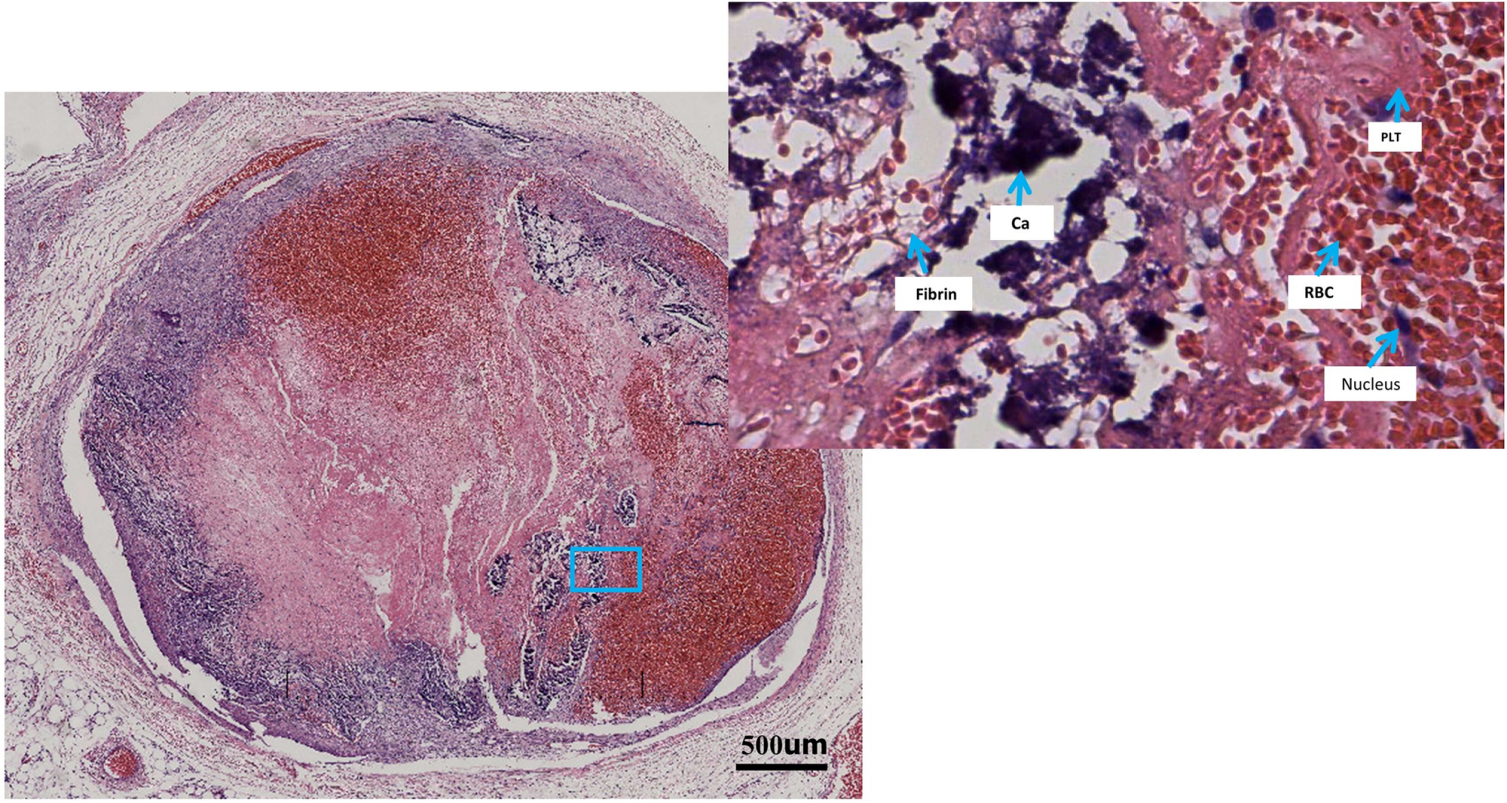
Pathological images of a thrombus cross-section scanned by an automatic pathological digitized scanning system (APDSS) showing clot construction in rabbit #27 on day 11 after induction. The left image shows the scanning image of the whole thrombus; the right image shows the enlarged part of the scanning image to show different thrombus components. A fuzzy boundary between the thrombus and the vessel wall at the 12 o’clock position and the new filling with fresh red cells in the left image was considered organization and subsequent recanalization. RBC, red blood cell; PLT, platelet trabecula; Ca, calcium salt deposition.

After the SWE measurements, three rabbits were randomly selected daily for euthanization via intravenous air injection. Immediately after death, the thrombi were exteriorized and trisected into the following three parts: head, body and tail. All parts were fixed in 10% neutral buffered formalin for 14 days, dried using alcohol solutions in increasing concentrations and finally embedded in paraffin. Before embedding, we selected the center of each part of thrombus as the final area of interest to ensure that all histological slices contained an area examined by SWE. Using a microtome, slices with a thickness of 4–6 μm were obtained and stained by hematoxylin and eosin (HE) according to a standard protocol. Each section was scanned using an automatic pathological digitized scanning system (APDSS, PRECICE 600x8, Unic, China) for image processing (Fig 3).

The scanned images of the thrombus were manually delineated using Photoshop (7.0, Adobe, Cal, America). Then, the images were exported to MATLAB 7.0 (MathWorks, Natick, MA, USA). The red, pink and blue image components were isolated and calculated, and their relative percentages were obtained [17]. A red content parameter (Rr) representing red blood cells (RBCs) and platelet trabecula was determined by nonlinear weighting of the fractional intensity of the red component [16, 18]. Similarly, a pink parameter (Rp) representing fibrin was determined by the pink component, and a blue parameter (Rb) representing the nucleus and calcium salt deposition was calculated as 1 minus Rr and Rp. We identified the percentage of the different color components in the sections to obtain the relative quantification of the pathological compositions in the different stages. Histological analysis was assessed by two experienced pathologists (more than 5 years of experience) who were blinded to the SWE measurements. A total of 182 sections were obtained in our study and subsequently assessed via image analysis as described above.

## Results

Of the 56 rabbits investigated in the present study, seven were excluded from the study; two died after injection of the anesthetics before surgery; one died during the course of the experiment; two did not develop a thrombosis; and two experienced autolysis. The remaining rabbits were in good condition before euthanasia and had developed an IVC thrombosis for study.

### Elasticity tendencies over the 2-week period

**Fig 4:**
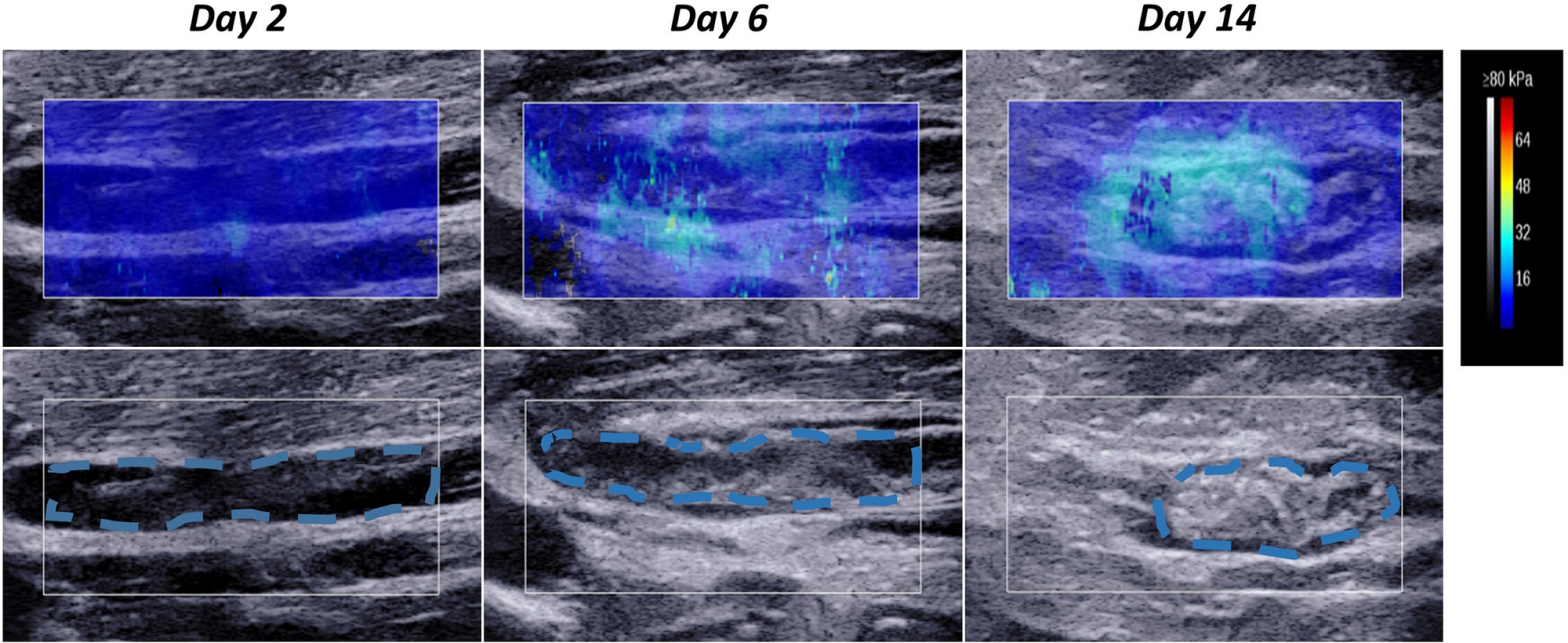
Ultrasound variation of the ROI in the thrombus of rabbit #35 on day 2 (left), day 6 (middle) and day 14 (right). The lower row shows gray-scale ultrasound changes in the ROI, the thrombi in the ROI are traced by the blue dashed line; the upper panel shows the shear wave elastography (SWE) changes in the ROI. The vein under analysis and the adjacent tissue are indicated by a solid-line rectangular frame. Thrombus hardening correlates with a gradual color change from dark blue to deep red in the SWE images. The Young's modulus (YM) values were 3.15 kPa (day 2), 7.20 kPa (day 6) and 12.39 kPa (day 14).

Fig 4 presents the images for the three different stages after thrombi induction of rabbit #35 on day 2 (left), day 6 (middle) and day 14 (right). First, the gray-scale ultrasound (lower panel) shows that the size was reduced and gradual heterogeneity in the thrombus appearance occurred as the thrombi matured from day 2 to day 14. On day 2, the thrombus had a homogeneous hypoechoic appearance and the boundary with the vessel wall was extremely clear. On day 6, the thrombus showed a non-uniform medium echo with certain hyperechoic areas and the boundary with the vessel wall grew closer. On day 14, the thrombus significantly shrank and showed hyperechoic areas and more obvious heterogeneity as the region fused with the vascular wall and the boundary could no longer be distinguished.

Second, the upper panel shows the changes in elasticity variations with thrombus aging. The elasticity differences in the ROI among the three thrombi show a color spectrum from dark blue (day 2) to light blue (day 14). On day 2, the color appeared as uniform dark blue, which indicates a low YM according to the color bar. On day 6, the color was mostly dark blue with some light blue. On day 14, the dominant color was bright blue and the heterogeneity became more obvious, which was consistent with the gray-scale ultrasound. Correspondingly, the mean YM values were 3.15 kPa, 7.20 kPa, and 12.39 kPa on day 2, day 6 and day 14, respectively.

### Elasticity trend in the three thrombus parts during the 2-week period

**Fig 5:**
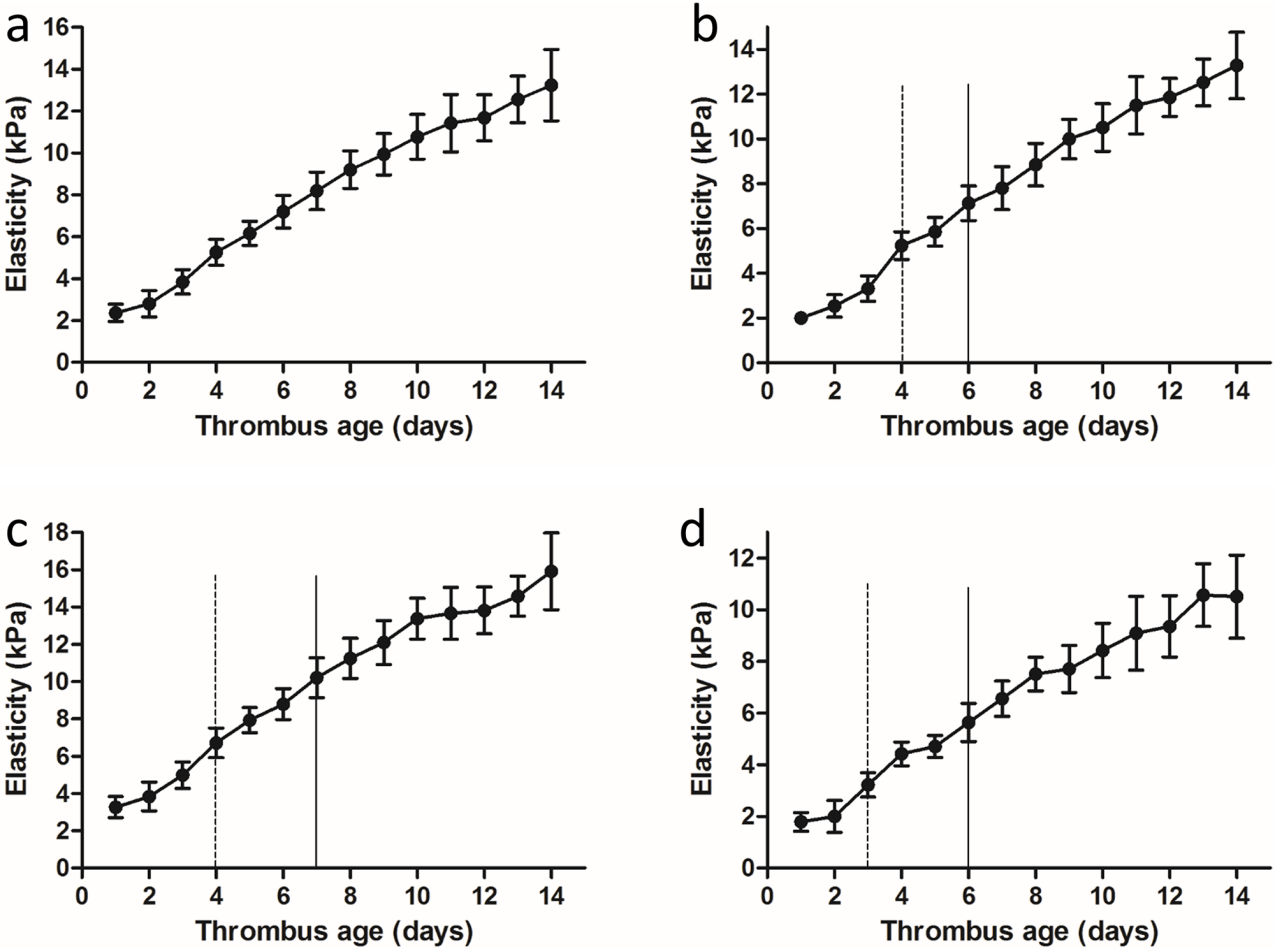
Average YM curves of the whole thrombus (a) and the three parts (b: head; c: body; d: tail) over the 2-week monitoring period starting at thrombus induction. The most obvious transition points are marked by solid and dashed lines. Data are expressed as the mean ± SD for the detection of YM in vivo at one time point.

Considering the different course of formation and evolution of the three parts of the thrombus (head, body, tail), the elasticity of each was measured individually (Fig 2). In this study, the mean YM value of three parts represented the elasticity of the whole thrombus. The DVT matured at 2 weeks (Fig 5), and the elasticity of the whole thrombus (a) and its three parts (b: head; c: body; d: tail) revealed an increasing trend, with the YM values varying from 2.36 ± 0.41 kPa to 13.24 ± 1.71 kPa; 2.01 ± 0.28 kPa to 13.29 ± 1.48 kPa; 3.27 ± 0.57 kPa to 15.91 ± 2.05 kPa; and 1.79 ± 0.36 kPa to 10.51 ± 1.61 kPa, respectively. The deviation of elasticity gradually increased, especially after day 6 of thrombus induction, thus indicating more obvious heterogeneity among different clots as the thrombi matured.

Differences were observed among the four elasticity curves: the elasticity of the head obviously increased on days 4 and 6; the elasticity of the body increased on days 4 and 7; and the elasticity of the tail increased on days 3 and 6. However, these trends were not obvious throughout the entire thrombus.

### Pathology variation in different stages

**Fig 6:**
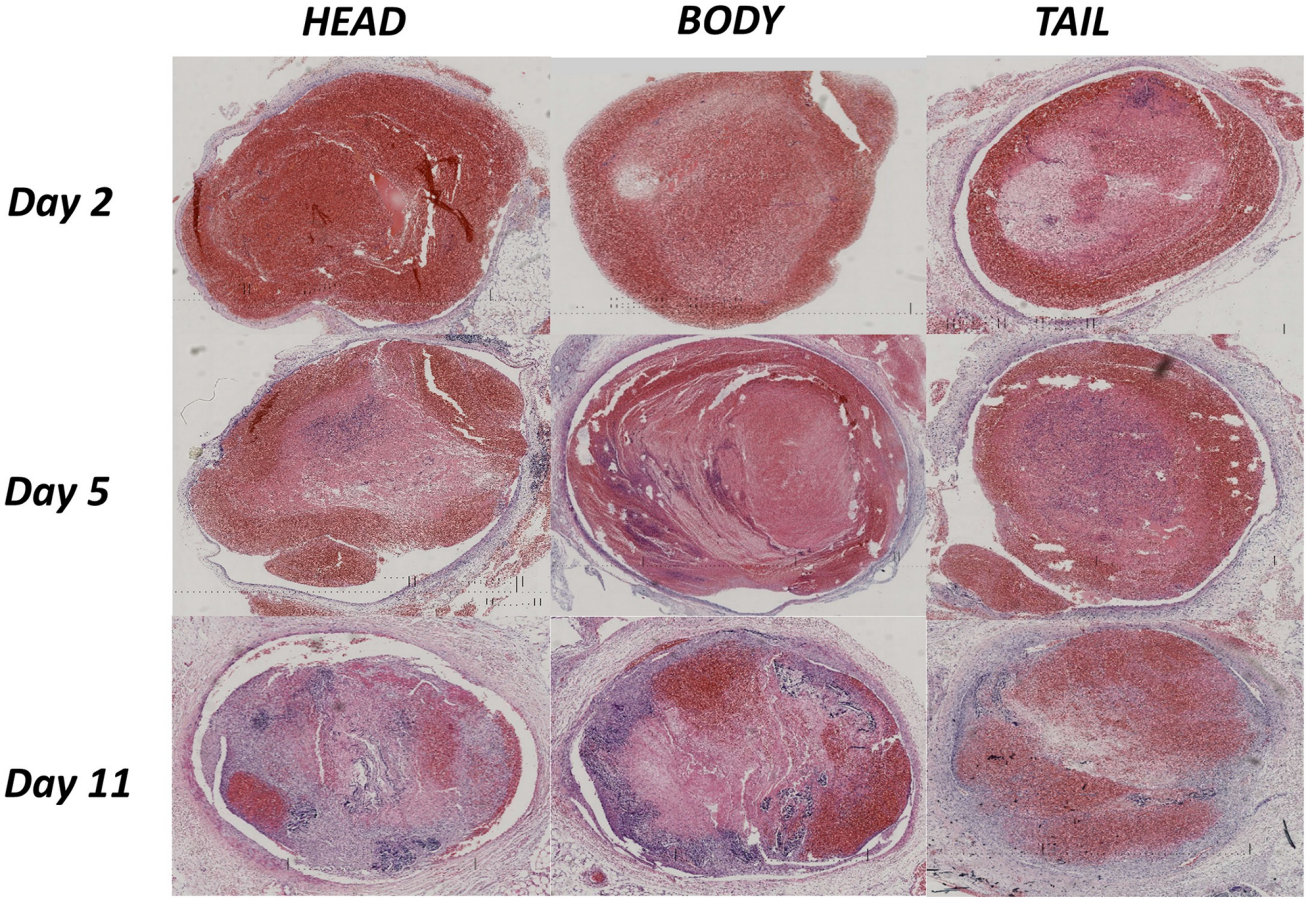
Pathological scanning images of different thrombus stages on day 2 for rabbit #8, day 5 for rabbit #44 and day 11 for rabbit #27. Each thrombus was divided into the following three parts: head (left), body (middle) and tail (right). Large differences were observed in the thrombus components both between the thrombi on different days and within a single thrombus on the same day.

Thrombi from rabbit #8 (day 2), #44 (day 5) and #27 (day 11) were selected, and the pathological scanning images of the three parts (head, body, tail) are shown in Fig 6. With DVT aging, the compositions of the thrombus appeared to change dynamically, with the deposition of fibrin and calcium salts showing a gradually increasing trend and the RBCs and platelet trabecula showing a corresponding decreasing trend. The boundary between the thrombus and the vessel wall was extremely clear on day 2, blurry in some parts on day 5, and could not be differentiated on day 11, which indicated an organization process with thrombus maturity. Interestingly, the RBCs and platelets aggregated again in the thrombus on day 11, which was considered to represent recanalization after organization.

The heterogeneity of thrombus components became more obvious with DVT maturity. On day 2, the three parts of the thrombus showed good homogeneity and an overwhelming content of RBCs and platelets. Obvious heterogeneity among the thrombus compositions could be observed on day 5 and became more significant on day 11, and a dramatic increase in calcium salt deposition was observed. The QPIA further exhibited quantitative variations in thrombus composition in the three parts at different stages (Table 1). The gray-scale values of the thrombus composition were similar on day 2, with Rp as the main component. The Rp content significantly increased on day 5, and the Rb content continually increased until day 11, with significant heterogeneity observed among the three parts.

**Table 1:** Quantitative variations in thrombus composition of the head, body and tail at different stages on day 2, day 5 and day 11.

Age (day)	Head			Body			Tail		
	Rr	Rp	Rb	Rr	Rp	Rb	Rr	Rp	Rb
	(%)	(%)	(%)	(%)	(%)	(%)	(%)	(%)	(%)
2	97.02	2.47	0.51	95.75	3.54	0.71	92.63	6.27	1.10
5	81.72	16.49	1.79	80.22	19.30	0.48	77.44	16.38	6.18
11	47.39	42.17	10.44	50.70	34.54	14.76	74.20	16.37	9.43

Rr, region of red containing RBCs and platelet trabecula; Rp, region of pink containing fibrin; Rb, region of blue containing the nucleus and calcium salt deposition.

### Pathologic variation tendencies over the 2-week period

**Fig 7:**
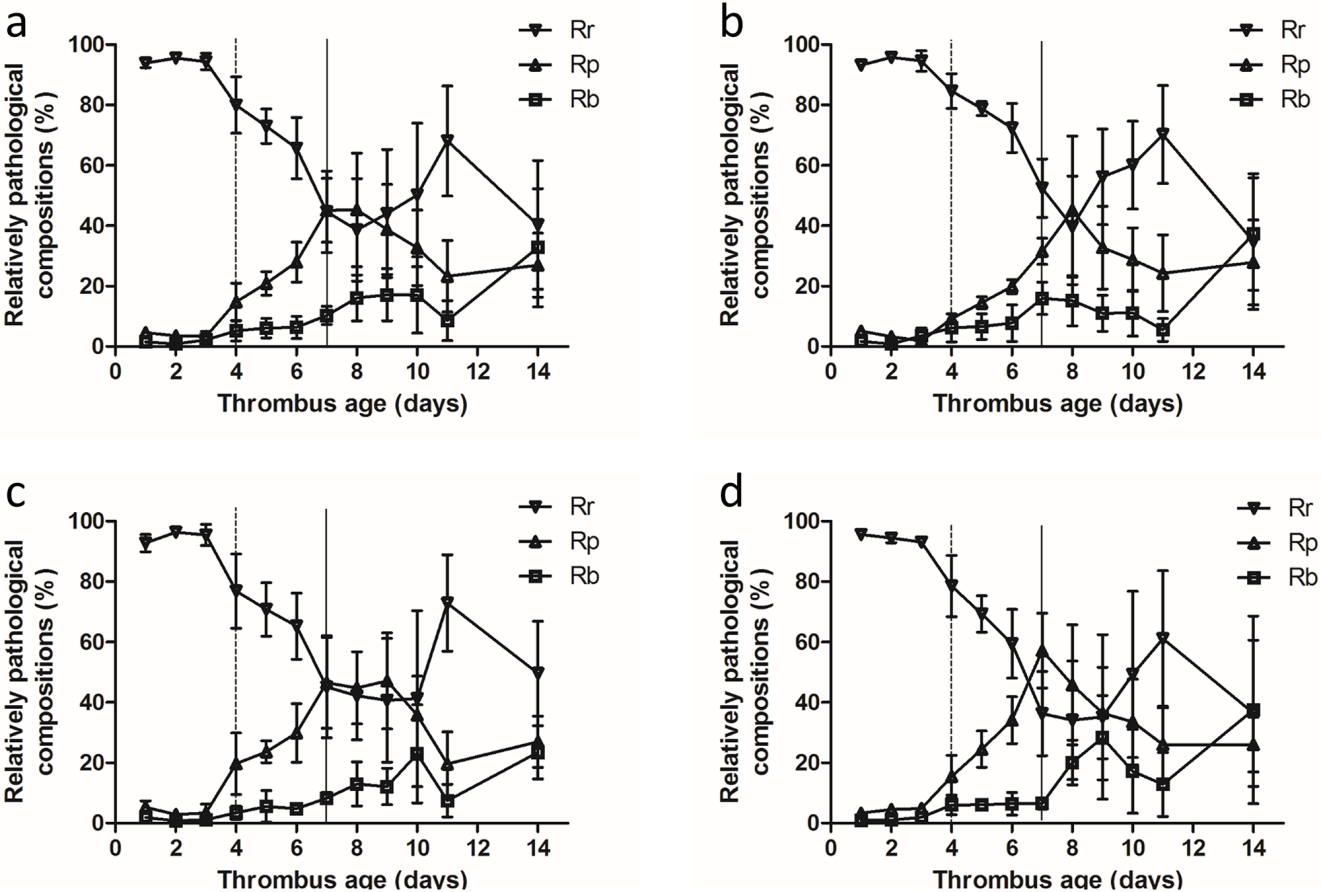
Trends in variations of the thrombus composition for the whole thrombus (a) and the three parts (b: head; c: body; and d: tail). Data are expressed as the mean ± SD, n = 3 cases at each corresponding examination point. All trends became more obvious on day 4 (dashed lines) and day 7 (solid lines). Rr represents the components of red blood cells and platelet trabecula, Rp represents fibrin and Rb represents the nucleus and calcium salt deposition.

We calculated the mean values of the Rr, Rp and Rb contents in the three parts to represent their relative contributions to the whole thrombus. Fig 7 shows the trends gray-scale value variations of the whole thrombus (a) and the three parts (b: head; c: body; and d: tail), and they all exhibited similar trends. Before day 10, Rr showed a gradual decreasing trend and Rp and Rb showed corresponding increasing trends. Significant changes occurred on days 4 and 7. On days 1–3, significant changes were not observed in the composition of the thrombus, which displayed good homogeneity during this stage. The pathological components changed significantly on days 4 and 7, which may represent transition points for thrombus staging. The SD of the gray-scale values were small on days 1–3, which indicated good homogeneity among the different thrombi parts. The deviation gradually increased from day 4 and became more obvious after day 7. We concluded that obvious heterogeneity occurred among the different thrombi starting on day 4, and the difference was more significant after day 7.

The gray-scale values changed significantly again after day 10, and Rr gradually increased while Rp and Rb gradually decreased. According to the pathology image, the thrombi were clearly filled with fresh RBCs and platelets in the later stage. We inferred that the thrombi organization and subsequent recanalization caused the changes in the corresponding thrombi composition. Additionally, the recanalization of the three parts occurred at different times: on day 9 in the head; on day 10 in the tail; and on day 11 in the body. In summary, the recanalization first appeared at the two sides of the thrombus and appeared later in the body (Fig 7).

### Heterogeneity analysis of the thrombi

**Fig 8:**
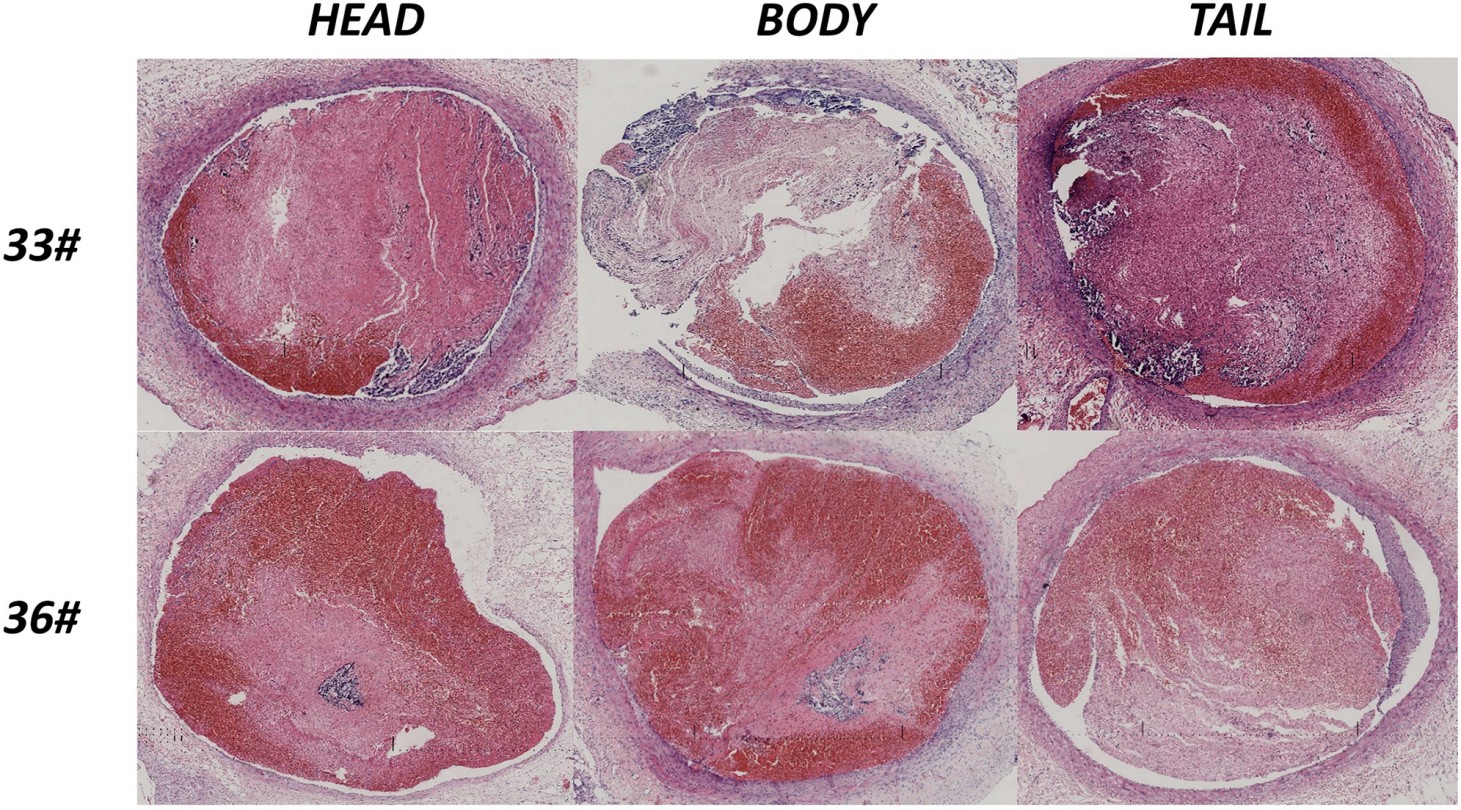
Heterogeneity of the pathological results between two rabbits (#33 and #36) on day 8. Each thrombus was divided into the following three parts: head (left), body (middle) and tail (right). Large differences were observed in the components both between two thrombi and within the three different parts of a single thrombus.

The pathological results above indicated that significant differences occurred among the different thrombi after day 3 of induction. Two clots from rabbits #33 and #36 were selected on day 8 to explore the diversity between different thrombi (Fig 8). Large differences in the pathological components were observed between the thrombi of these two animals. Overall, the deposition of fibrin and calcium salts played a dominant role in the thrombus of rabbit #33, whereas the overwhelming components were red cells, platelet trabecula and fibrin in rabbit #36, and the three parts of the thrombi showed greater homogeneity in rabbit #36 than rabbit #33. The two thrombi in these animals may not have been at the same stage, despite the animals being the same age. The QPIA quantitatively showed the different components in the two thrombi (Table 2). Within the same thrombus, the compositions also differed among the head, body and tail. Therefore, obvious heterogeneity occurred between and within the thrombi.

**Table 2:** Quantitative variations in thrombus composition of different parts in rabbits #33 and #36 on day 8.

Models	Head			Body			Tail		
	Rr	Rp	Rb	Rr	Rp	Rb	Rr	Rp	Rb
	(%)	(%)	(%)	(%)	(%)	(%)	(%)	(%)	(%)
33#	19.65	68.59	11.76	29.98	46.75	23.27	11.59	60.92	27.49
36#	38.87	55.62	5.51	33.92	58.41	7.67	31.25	58.78	9.97

Rr, region of red containing RBCs and platelet trabecula; Rp, region of pink containing fibrin; Rb, region of blue containing the nucleus and calcium salt deposition.

## Discussion

The treatment modalities for DVT differ according to the stage of DVT. Thus, accurate clinical staging is important for guiding clinical treatment, especially for identifying acute thrombi. Certain treatments, such as anticoagulant medication or surgical thrombectomy, can have dramatic efficacy during this period [15].

In the present study, we set up a stenosis-induced DVT model and applied a QPIA to observe the dynamic variations in the thrombus components. Good homogeneity was observed in the thrombi on days 1–3 after induction. Changes in the thrombus components were observed starting on day 4, and a closer relationship with the vein wall and obvious heterogeneity among the thrombi were also observed. These changes became more significant after day 7. Calcium salt deposition increased dramatically, and organization and recanalization could also be observed (especially after day 10). These findings and the results of a previous report indicate that day 7 is a likely transition point between acute and chronic thrombus [14]; thus, we can speculate that days 4 and 7 may represent separate transition points between acute and sub-acute and sub-acute and chronic thrombi in rabbits.

The elasticity was continuously monitored by SWE, and the results indicated that the YM value increased with thrombus maturity. This trend was consistent with the changes in fibrin and calcium salt deposition in the pathological composition, which was related to the increased hardness of the thrombus[19]. The elasticity images also partially showed the dynamic hardness changes of blood clots and their relationship with the vein wall. These findings reveal that quantitative SWE can reflect the presence and dynamic evolution of DVT. In addition, continuously monitoring elasticity using SWE in the same part of the thrombus may be helpful for identifying the transition points for acute, sub-acute and chronic thrombus. The YM value increased significantly in the head on days 4 and 6, in the body on days 4 and 7, and in the tail on days 3 and 6; however, these changes were not observed in the whole thrombus. Therefore, the transition point for staging could not be found using the thrombus as a whole or by using random positions for elasticity measurement, and only continuous dynamic detection using SWE at specific locations was able to identify these points. In addition, previous study also indicated that dynamic US elasticity imaging could also be helpful for locating DVT recurrence in a timely manner [20].

The elasticity changes in the body of the thrombus exhibited greater consistency with the pathological analysis by QPIA compared with changes in the head and tail. This finding may be related to the differing levels of contact with blood in these three parts. The coagulation and fibrinolytic system cause continuous absorption and reformation of the thrombus, especially in the tail. The body was generally isolated from the blood flow and was therefore more stable. Thus, we suggest that the body is more suitable for thrombus staging by SWE.

As mentioned above, good consistency was observed between the findings on SWE and pathological variations. However, certain discrepancies were observed, such as the different changes after day 10 and the different transition points of the head and tail. Possible explanations for these discrepancies are listed as follows. First, the measurement conditions were different. The pathological analysis of the composition of blood clots was performed ex vivo in principle. However the SWE analysis of elasticity was performed in vivo, the influence of the circumferential vessel walls and surrounding tissue was difficult to avoid [15]. Additionally, the elasticity of the vessel walls and surrounding tissue also changed with thrombus aging [21]. Second, the significant heterogeneity between and within the thrombi may affect the thrombus staging. As the thrombus matured, the different thrombi displayed different evolutionary processes, and different compositions and evolutions were observed in the different parts, even within the same thrombus, which is consistent with the significant heterogeneity observed in previous studies [19, 22]. Another possible reason for the discrepancies is related to the statistical analysis, which showed that the accuracy of staging estimations via ultrasound elasticity was within 0.8 days, with the worst estimation showing an error of 1.7 days [10]. Therefore, a 1 to 2 day error in the predictions of the transition point could be acceptable.

In the present study, we systematically investigated the pathological changes in different parts of a DVT at different stages, and we examined their relationship with the findings on SWE. As the thrombus matured, the variation of elasticity quantified by the SWE analysis was consistent with the pathological composition, which confirmed the value of SWE for staging DVT. In addition, significant heterogeneity was observed between and within the thrombi, which may affect thrombus staging.

Our study also had certain limitations. First, we quantified the pathological compositions of DVT using QPIA, which may cause discrepancies with the actual pathological composition. Second, obvious recanalization occurred with thrombus aging, especially after day 10. The QPIA reflected all components in the vessels rather than the thrombi themselves, which may influence the accuracy of staging. Moreover, this finding could explain why the trends in SWE were not consistent with the pathology after day 10, which is consistent with the results reported by Xie et al. [23]. Third, we trisected the thrombus into the following three parts: head, body and tail. There might exist some discrepancies with the actual pathological structures. Fourth, previous studies have reported that the composition of nuclear cells in the vein wall vary at different stages [24]. However, the infiltration of nuclear cells into the thrombus was not mentioned. In our QPIA analysis, Rb reflected the total gray level of the blue parameter and could not distinguish the nuclear cells from calcium salt depositions. Therefore, a more effective method that can accurately reflect the variation of pathological components is needed and worthy of further research.

## Conclusions

An accurate estimate of the stage and maturity of DVT is of great importance for determining the proper therapeutic management. For the first time, we systemically studied the relationship between variations in the pathological components and the findings of the QPIA and SWE. SWE is a non-invasive method that can reflect the changes in the pathological components that occur as the thrombus matures. Continuous dynamic measurements at specific parts could help with thrombus staging, and the body is the optimal part for examination. Days 4 and 7 after thrombus induction may represent transition points for acute, sub-acute and chronic thrombi in rabbit models, and heterogeneity observed between and within thrombi may represent an important factor related to thrombus staging.

## Supporting information

S1 Table Ultrasound measurement of thrombus in two weeks after induction in the head, body and tail parts. (XLSX)

S2 Table Quantitative variations in thrombus composition of the head, body and tail parts in two weeks after induction. Rr, region of red containing RBCs and platelet trabecula; Rp, region of pink containing fibrin; Rb, region of blue containing the nucleus and calcium salt deposition.
(XLSX)

## Abbreviations

APDSS: automatic pathological digitized scanning system
DVT: deep venous thrombus
IVC: inferior vena cava
kPa: kilopascals
PE: pulmonary embolism
QPIA: quantitative pathologic image analysis
RBC: red blood cell
ROI: region of interest
SD: standard deviation
SSI: supersonic shear wave imaging
SWE: shear wave elastography
US: ultrasound
